# Low-dose vasopressin infusion results in increased mortality and cardiac dysfunction following ischemia-reperfusion injury in mice

**DOI:** 10.1186/cc7930

**Published:** 2009-06-23

**Authors:** Toonchai Indrambarya, John H Boyd, Yingjin Wang, Melissa McConechy, Keith R Walley

**Affiliations:** 1 Critical Care Research Laboratories, Heart + Lung Institute, University of British Columbia, 166 – 1081 Burrard Street, Vancouver, British Columbia, V6Z 1Y6, Canada

## Abstract

**Introduction:**

Arginine vasopressin is a vasoactive drug commonly used in distributive shock states including mixed shock with a cardiac component. However, the direct effect of arginine vasopressin on the function of the ischemia/reperfusion injured heart has not been clearly elucidated.

**Methods:**

We measured left ventricular ejection fraction using trans-thoracic echocardiography in C57B6 mice, both in normal controls and following ischemia/reperfusion injury induced by a one hour ligation of the left anterior descending coronary artery. Mice were treated with one of normal saline, dobutamine (8.33 μg/kg/min), or arginine vasopressin (0.00057 Units/kg/min, equivalent to 0.04 Units/min in a 70 kg human) delivered by an intraperitoneal micro-osmotic pump. Arterial blood pressure was measured using a micromanometer catheter. In addition, mortality was recorded and cardiac tissues processed for RNA and protein.

**Results:**

Baseline left ventricular ejection fraction was 65.6% (60 to 72). In normal control mice, there was no difference in left ventricular ejection fraction according to infusion group. Following ischemia/reperfusion injury, AVP treatment significantly reduced day 1 left ventricular ejection fraction 46.2% (34.4 to 52.0), both in comparison with baseline and day 1 saline treated controls 56.9% (42.4 to 60.2). There were no significant differences in preload (left ventricular end diastolic volume), afterload (blood pressure) or heart rate to account for the effect of AVP on left ventricular ejection fraction. The seven-day mortality rate was highest in the arginine vasopressin group. Following ischemia/reperfusion injury, we found no change in cardiac V1 Receptor expression but a 40% decrease in Oxytocin Receptor expression.

**Conclusions:**

Arginine vasopressin infusion significantly depressed the myocardial function in an ischemia/reperfusion model and increased mortality in comparison with both saline and dobutamine treated animals. The use of vasopressin may be contraindicated in non-vasodilatory shock states associated with significant cardiac injury.

## Introduction

With the increasing medical complexity of the critically ill, shock due to a combination of vasodilation and cardiac dysfunction is increasingly frequent. Two common clinical examples of this are first, vasodilation following cardiopulmonary bypass surgery and, second, the cardiac dysfunction during septic shock. These mixed shock conditions are routinely treated with intravenous fluids plus inotropes combined with a vasopressor such as norepinephrine or arginine vasopressin (AVP). AVP is a vasopressor commonly used in intensive care units and cardiac surgical units due to its efficacy in restorating blood pressure [1-6]. The effects of AVP are mediated via vasopressin 1 receptors (V1R; predominantly vascular), vasopressin 2 receptors (V2R; predominantly renal), vasopressin 3 receptors (V3R; predominantly central), and the oxytocin receptors (OTR) [7]. In addition, vasopressin blocks K_ATP _channels [8] and potentiates the effect of adrenergic agents [9]. Vascular V1R appear to mediate the majority of effects of vasopressin in reversing vasoplegia and catecholamine tolerance [4,10].

In healthy individuals, AVP administration at low doses (<0.04 Units/minute) has little effect on blood pressure. However, there are multiple reports of increased blood pressure responsiveness to low-dose AVP in both septic shock and distributive shock after cardiopulmonary bypass surgery [7,11]. Consequently, low-dose AVP has been increasingly used to treat these disorders [1-3,12-17].

Despite its widespread use, there remains considerable uncertainty regarding its cardiac effects. When studied at the high doses (0.1 to 1 Unit/minute) previously used for mesenteric vessel constriction in gastrointestinal bleeding [18], deleterious effects of AVP on myocardial performance were reported including coronary vasospasm [19-21]. At these high doses, AVP may also impair indices of ventricular contraction and relaxation without overt global ischemia [22]. In addition, the baroreflex mediated via V1R might cause bradycardia and direct cardiac suppression [23,24]. Although the most highly expressed vasopressin receptor in the heart is V1R, the other receptors are physiologically active. Gene transfer of V2R into failing myocardium increases cardiac contractility [25,26], while OTR mediates a calcium-dependent vasodilatory response via stimulation of the nitric oxide pathway in endothelial cells [27]. OTR stimulation also results in release of atrial natriuretic peptide from the heart [28,29].

Clinically, there are conflicting reports on the effect of AVP on cardiac function. In some series, AVP infusion has been reported to decrease cardiac output [28,30,31]. Others have observed a dramatic restoration of blood pressure without a decrease in stroke volume or other measures of cardiac function [2,30,32,33]. The clinical observation that AVP increases mean arterial pressure in patients with shock is uniform across these studies, so interpreting any direct effect on myocardial contractility must be done with caution as alterations in afterload have a significant impact on measures of cardiac performance.

The uncertainty as to the *in vivo *action of AVP on the heart provides the rationale for this study. Further, as the use of AVP moves into the mainstream [1,12], it is important to understand its cardiac effects both on the normal heart and in the injured or ischemic heart. We chose a model of subacute heart failure without overt shock as the direct *in vivo *effects of AVP on contractility are extremely difficult to distinguish from indirect effects due to changes in afterload (blood pressure). In this study, we used a mouse model of ischemia/reperfusion (I/R) induced heart failure to compare the effect of continuous infusion of AVP with saline control (SL) or standard inotropic therapy (dobutamine (DOB)) on cardiac function in mice. We assessed cardiac function using trans-thoracic echocardiography, and in parallel experiments used intra-arterial pressure measurements to determine whether cardiac function was influenced by changes in systemic blood pressure.

## Materials and methods

These experiments were approved by the UBC Animal Care Committee and conform to Canadian and National Institutes of Health guidelines regarding animal experimentation. All experiments were conducted in 10- to 14-week-old male C57B6 mice as a control and in mice following I/R injury induced by one hour ligation of the left anterior descending coronary artery (LAD; see below). Intraperitoneal pumps (1 μL/hour for 72 hours, Alzet micro-osmotic pump, Alza Corporation, Palo Alto, CA, USA) delivered normal saline (SL control), DOB at 8.33 μg/kg/minute, or arginine vasopressin at 0.00057 Units/kg/minute (equivalent to 0.04 Units/minute in a 70 kg human; AVP treatment). AVP levels in rodents and humans are similar, while in rodents the intraperitoneal route of administration for AVP increases plasma AVP levels in a manner very similar to intravenous dosing in humans [34,35]. At least five mice per time point in each group were studied.

### Ischemia-reperfusion of the LAD

An open-chest model of I/R using ligation and reperfusion of the LAD was modified from Michael and colleagues [36]. Mice were anesthetized using ketamine (75 mg/kg) and xylazine (10 mg/kg) in order to facilitate endotracheal intubation using a 22 Gauge catheter. Thereafter, deep anesthesia was maintained with 1 to 2% isoflurane. Ventilation was controlled using Mouse Ventilator (Model 687, Harvard Instruments, Holiston, MA, USA) with a tidal volume of 0.5 mL and a respiratory rate of 120 breaths/minute. After a left thoracotomy was performed at the level of the second or third intercostal space, the LAD was identified and a 6-0 polypropylene suture was placed around the LAD. Occlusion of the LAD was accomplished by pulling the suture ends through a small piece of PE-50 tubing and occlusion was confirmed by discoloration of the anterior left ventricle wall.

Following one hour of ischemia the ligature was released to allow reperfusion, which was visualized. Following the thoracotomy wound closure, the intraperitoneal pump (see above) was implanted into the peritoneal cavity. Intra-operatively, 1 mL of normal saline was injected subcutaneously for volume resuscitation and subcutaneous buprenorphrine for pain control were given. After recovery and resumption of spontaneous ventilation, mice were extubated.

### Myocardial function evaluation

Left ventricular ejection fraction (LVEF) was used to measure cardiac function at baseline, day 1 and day 3 post I/R. Transthoracic echocardiography using a Vevo 770 cardiac ultrasound (Visualsonics, Toronto, Canada) while anesthetized with 1 to 2% inhaled isofluorane. Left ventricular internal diameter at end-systole and end-diastole from Short Axis 2D views at the level of the papillary muscles were identified and used for measurement of LVEF using the manufacturer's software. All echocardiographs were performed by the same qualified investigator (TI), and quality control was ensured by the other investigator (JB) blinded from treatment group.

### Direct blood pressure measurement

As arterial catheterization is a terminal procedure, separate mice were anesthetized in the same way and a number 2 French micromanometer catheter (Mikro-tip SPR-838, Millar Instruments Inc., Houston, TX, USA) was advanced via the carotid artery into the ascending aorta to measure blood pressure. The heart was excised, frozen in liquid nitrogen, and stored at -80°C for subsequent study.

### Quantitative real-time PCR

Total RNA was extracted from frozen heart samples using Trizol (Invitrogen, Carlsbad, CA, USA) as per the manufacturer's instructions. RNA was obtained from either I/R injured hearts or control, non-injured heart. RNA 1 μg was treated with DNAse I Amplification Grade (Invitrogen, Carlsbad, CA, USA) and the product underwent quantitiative RT-PCR using M-MLV RT (Invitrogen, Carlsbad, CA, USA) followed by PCR amplification with Taq DNA Polymerase (Qiagen, Valencia, CA, USA). PCR was 40 cycles at 94°C for 15 seconds, 58°C for 30 seconds, and 72°C for 30 seconds. Primers were as follows, V1R forward; TCGTCCAGATGTGGTCAGTC, V1R reverse; AGCTGTTCAAGG-AAGCCAGT, V2R forward; CCTGGTGTCTACCACGTCTG, V2R reverse; GGTCTCGGTCATCCAGTAGC. OTR Forward; AGGAGCTGTTCTCAACCATC OTR Reverse; QPCR TGCAAACCAATCAATAGGCAC. SYBER green was used as the fluorescence indicator, which represented quantity of amplicon production with PCR cycle (Ct value). All quantitative RT-PCR reactions were run in triplicate and an average Ct value was calculated for each PCR condition. Fold change of Ct value of each sample was calculate using glyceraldehyde-3-phosphate dehydrogenase (GAPDH) as a background control.

### Western blot for OTR

A 20 μg sample of each protein was mixed with equal volumes of SDS reducing buffer (62.5 mmol Tris l–1, pH 6.8, 2% (w/v) SDS, 10% (v/v) glycerol, 100 mmol dithiothreitol l–1, 0.05% (w/v) bromophenol blue) and incubated in a boiling waterbath for five minutes before loading. Using the discontinuous buffer system SDS-PAGE, proteins were separated according to size on 10% polyacrylamide gels and electroblotted on to nitrocellulose membranes. After blocking non-specific antigens with 5% (w/v) skim milk for one hour, western blots were probed with rabbit's anti-OTR immunoglobulin (Santa Cruz Biotechnology, California, USA), dilute 1:2000 in 5% (w/v) BSA and Tris-Buffered Saline Tween-20 at 4°C overnight. Using the Enzymatic Chemiluminescence (ECL, Amersham™, GE Healthcare, Buckinghamshire, UK) assay, anti-rabbit horseradish peroxidase molecule bound goat immunoglobulin was used as secondary antibody. The images of ECL reaction were obtained using Chemigenius2 with CCD camera (Syngene, Cambridge, UK). The densitometry was performed using imageJ 1.410 (National Institutes of Health, Maryland, USA).

### Data analysis

All graphical values are expressed as means ± standard error of the mean, and to provide more descriptive data in the results section we present data as median (inter-quartile range). In the case of unequal variance, groups were analyzed using Kruskal-Wallis one-way analysis of variance (ANOVA) on Ranks and subsequent multiple comparisons were performed using Dunn's Method. In groups with equal variance one-way ANOVA determined if differences existed, then pairwise multiple comparison procedures used the Holm-Sidak method. The analyses were performed using Sigmastat (SPSS, Chicago, IL, USA), and statistical significance was set at *P *< 0.05. Kaplan Meier survival was used to demonstrate the survival rate of each treatment group, and log rank test was used to test for differences between groups.

## Results

### Vasopressin significantly reduces left ventricular ejection fraction following I/R but has no effect in intact mice

**Figure 1:**
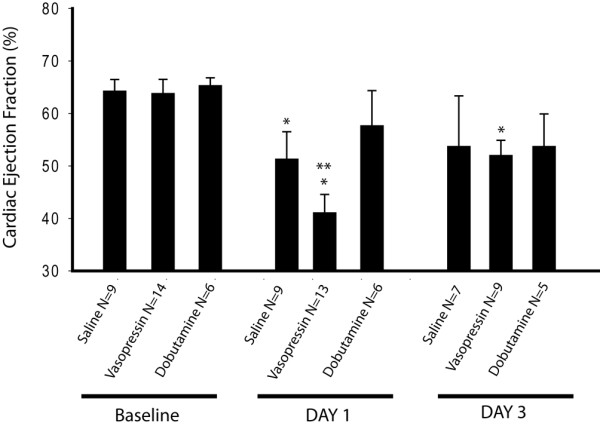
Cardiac function as assessed by 2D ECHO at baseline and following I/R of the LAD. The baseline (normal) left ventricular ejection fraction (LVEF) obtained from 2D short axis M-mode left ventricular internal diameter trace was 67.52 ± 1.8%, 63.48 ± 2.9%, and 65.47 ± 2.4% in arginine vasopressin (AVP; n = 14), normal saline solution (SL; n = 9), and dobutamine (DOB; n = 6), resepctively. Following ischemia/reperfusion (I/R) injury, AVP treatment significantly reduced day 1 LVEF (41.1 ± 3.4%) in comparison with SL control (51.6 ± 4.3%), while both group had significant reductions in LVEF vs baseline. DOB mitigated the decrease in LVEF (57.7 ± 6.7%) day 1 post I/R. LVEF measured at day 3 demonstrated improvement in all groups; however, mice receiving AVP remained significantly lower than baseline. * *P *< 0.05 vs baseline, ** *P *< 0.05 vs SL-treated mice. Results are present as means ± standard error of the mean. LAD = left anterior descending coronary artery.

The baseline (normal) LVEF obtained from 2D short axis M-mode left ventricular internal diameter trace was 65.6% (60 to 72; n = 29). In mice (n = 4 per group) who received intraperitoneal infusions but were not subjected to I/R of the LAD, there was no statistically significant difference in LVEF between SL controls at 62.7% (56.9 to 62.5), DOB 72.94% (73.9 to 56.0), and AVP treatment 54.73% (52.1 to 57.3). In mice subjected to I/R injury, AVP treatment significantly reduced day 1 LVEF to 46.2% (34.4 to 52.0) in comparison with both baseline and with day 1 SL control 56.9% (42.4 to 60.2), while DOB-treated mice did not demonstrate a significant reduction in day 1 LVEF compared with baseline 53.7% (47.0 to 61.7), as shown in Figure 1. In comparison to day 1, LVEF measured at day 3 demonstrated improvements in all groups; however, mice receiving AVP remained significantly lower than baseline.

### The decreased LVEF in vasopressin treated mice is due to altered contractility rather than through influencing heart rate, preload or afterload

**Figure 2:**
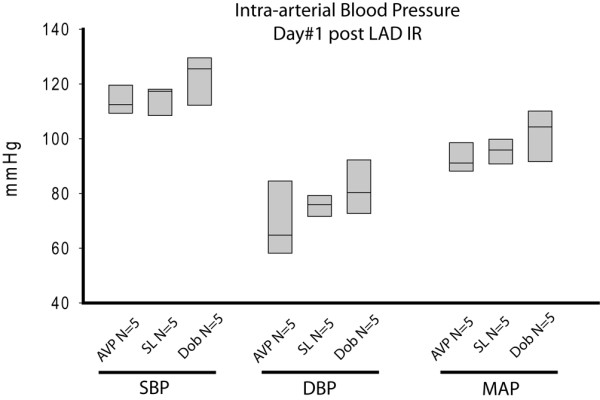
Intra-arterial blood pressure at day 1 following I/R of the LAD. Systolic blood pressure (SBP), diastolic blood pressure (DBP), and mean arterial pressure (MAP) are shown. Although there was no statistically significant differences in blood pressure, MAP trended to lowest in arginine vasopressin (AVP) group (n = 5) with a MAP of 89.7 ± 1.7 mmHg compared with 100.1 ± 6.0 in dobutamine (DOB; n = 5) and 94.8 ± 3.4 in normal saline solution (SL) control (n = 5). Results are present as means ± standard error of the mean. I/R = ischemia/reperfusion; LAD = left anterior descending coronary artery.

Baseline heart rate was similar in AVP, SL, and DOB groups respectively, with no statistically significant differences between groups. Following I/R of the LAD there was no statistical difference between groups at days 1 and 3 (Table 1). To assess left ventricular preload, we measured left ventricular end diastolic volume (LVEDV) using transthoracic echocardiography. Although there was a trend towards decreased LVEDV at day 1 and day 3 in all groups compared with their respective baseline values, there was no significant difference between AVP, SL, and DOB-treated mice at day 1 or day 3 after I/R (Table 1). Afterload was assessed through invasive measurement of systolic blood pressure, diastolic blood pressure, and mean arterial pressure are shown in Figure 2. Although there was no statistically significant differences in blood pressure, mean arterial pressure trended to lowest in AVP group with a mean arterial pressure of 91.1 mmHg (88.2 to 98.6) compared with 104.3 mmHg (91.6 to 110.1) in DOB and 95.9 mmHg (90.8 to 99.8) in SL controls.

**Table 1:** Baseline, day 1 and day 3 heart rate and left ventricular end diastolic volume post I/R injury and intraperitoneal pump implantation

**Group**	**Parameter**	**Baseline**	**Day 1 post I/R**	**Day 3 post I/R**
**Vasopressin** **n = 14**	**HR (bpm):** **LVEDV (uL):**	495 ± 18.1 67 ± 5	517.15 ± 15.67 58 ± 8	485.11 ± 34.94 60 ± 9
**Dobutamine** **n = 6**	**HR (bpm):** **LVEDV (uL):**	439 ± 28.7 64 ± 7	475.17 ± 32.35 55 ± 7	507.1 ± 50.68 55 ± 7
**Normal Saline** **n = 9**	**HR (bpm):** **LVEDV (uL):**	448 ± 15.1 69 ± 4	486.66 ± 16.29 60 ± 6	438 ± 32.10 58 ± 6

Heart rate (HR) was determined using limb lead echocardiography pads during echocardiogram, while left ventricular end diastolic volume (LVEDV) was determined using echocardiography. No significant differences in HR or LVEDV exist between groups infused with saline (n = 9), dobutamine (n = 6), or vasopressin (n = 14). Results are present as means ± standard error of the mean. I/R = ischemia/reperfusion.

### Vasopressin infusion results in higher mortality following I/R of the LAD than saline or dobutamine

**Figure 3:**
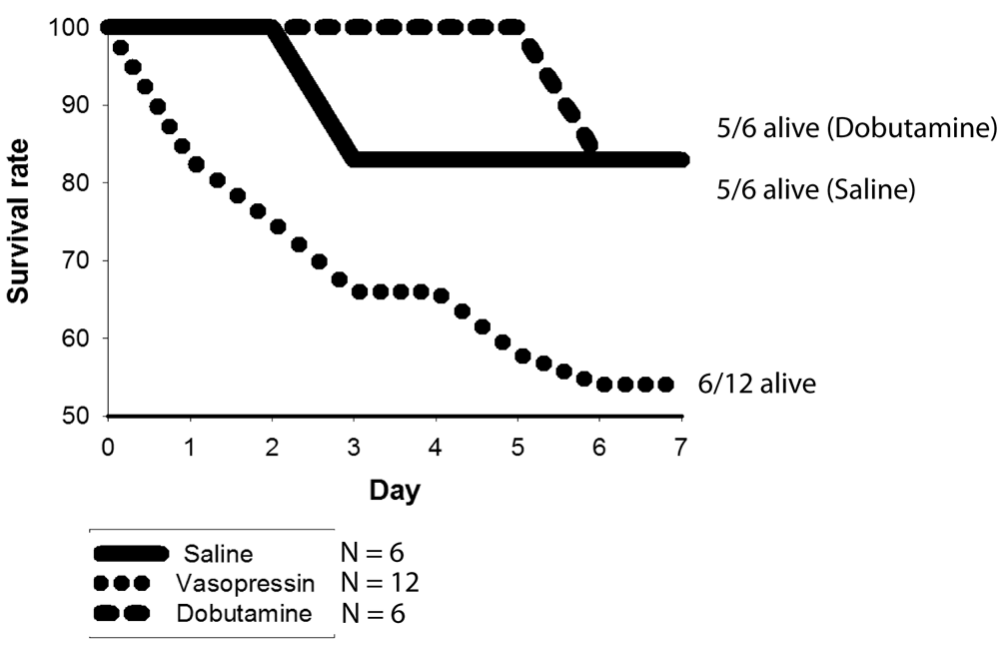
Kaplan Meier survival curve for mice in the three treatment groups. When compared with infusions of either saline (n = 6) or dobutamine (n = 6), vasopressin (n = 12) results in dramatically increased mortality. This difference begins as soon as day 1 following ischemia/reperfusion and persists throughout our seven day observation period.

When compared with infusions of either saline or DOB, vasopressin results in dramatically increased mortality (Figure 3). This difference begins as soon as day 1 following I/R and persists throughout our seven-day observation period. The mice were no different in appearance (grooming, temperature, activity level) according to infusion group, and in general appeared healthy during routine monitoring.

### Only V1R and OTR are expressed in the heart, I/R of the LAD results in changes in expression of OTR

**Figure 4:**
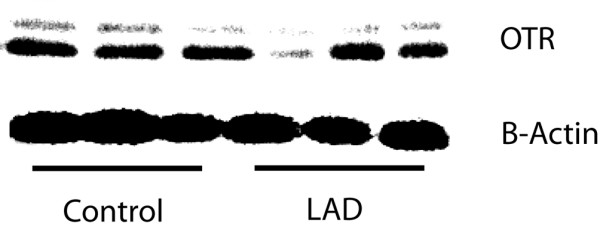
Western blot of left ventricular OTR levels at baseline and day 1 following I/R of the LAD. Left ventricular tissue was dissected and flash frozen for protein extraction both in control (baseline) animals and at day 1 following ischemia/reperfusion (I/R) of the left anterior descending coronary artery (LAD). We chose this timepoint as the enhanced physiologic effect (cardiac suppression) was observed by day 1. In those animals subjected to I/R of the LAD, oxytocin receptor (OTR) expression normalized to β-actin was reduced by 40% compared with controls.

Vasopressin has minimal effects on cardiac performance in intact animals, while I/R injury results in a dramatic suppression in cardiac ejection fraction when compared with saline infusion. We therefore verified whether this might be due to regulation of vasopressin receptor subtype in the heart as a result of I/R. Expression of V1R, V2R, and OTR in the heart was assessed in four mice per group at baseline and at day 1 following I/R of the LAD. Using RT-PCR, we found that normal hearts express only V1R and OTR, while V2R is not detectable. There was no change in V1R expression as a result of I/R injury, while OTR expression was reduced by 40% compared with controls (Figure 4).

## Discussion

The major finding of this study is that although continuous infusion of low-dose AVP (equivalent to 0.04 Units/minute in an average human) had no effect on hemodynamics or cardiac function in the resting state, following one hour of LAD I/R, AVP had a negative inotropic effect and seemed to increase early mortality. As previous studies have noted, AVP may exert cardiac suppressive effects through a variety of mechanisms[22-24,30,31], therefore, we went on to identify a potential mechanism behind this ischemia-induced cardiac sensitization to vasopressin.

Vasopressin is a peptide produced by the hypothalamus. Its effects are mediated through at least five specific receptors V1R, V2R, V3R, OTR, and purinergic receptors (P2R) [4,10]. V1R is the receptor thought to be primarily responsible for increased vascular tone because it mediates vasoconstriction in vascular smooth muscle. It has also been found to be expressed on cardiac myocytes and the kidney. V2Rs are found mainly in the renal collecting duct and are responsible for the antidiuretic effect of vasopressin. OTRs are found diffusely throughout the body and are thought to mediate vasodilation. Thus vasopressin is able to cause either vasoconstriction or vasodilation depending on the tissue specific distribution of V1R vs OTR and is able to enhance the effect of vasoconstrictor agents such as norepinephrine through mechanisms yet to be identified [9].

Although its mechanism of action on the vasculature is well understood, vasopressin has dose-dependent effects on both cardiac contractility and coronary arterial tone. It appears that at low doses vasopressin may act mainly through the P2R with a shifting of physiologic effect from coronary smooth muscle V1R-mediated vasoconstriction to P2R-mediated vascular endothelial vasodilation. At higher doses this relation is reversed with V1R-mediated coronary arterial vasoconstriction predominating, with a resultant drop in cardiac output. Due to safety concerns at higher doses, most clinical data relating to direct cardiac effects are using low doses of vasopressin (≤ 0.04 Units/minute), often in conjunction with inotropes. Patients with vasodilatory shock increase systemic vascular resistance twofold, while only diminishing cardiac output by 14% in response to vasopressin – indirect evidence of some positive inotropy [37]. Similarly, when co-infused with the phosphodiesterase inhibitor milrinone in patients with advanced heart failure, vasopressin resulted in increased vascular tone and blood pressure with no resultant change in cardiac output [38]. In hypotensive post-cardiotomy patients who remain in shock despite catecholamine infusions, the addition of low-dose vasopressin resulted in a significant increase in left ventricular work index and a decrease in vasopressor use, inotrope usage, and heart rate [2]. It is of great interest to the clinician that the hemodynamic effects of vasopressin are potentiated by the shock state, because in normal subjects vasoconstriction only occurs at high doses, while fluid unresponsive shock confers a powerful vasopressor effect at low doses. This may reflect an acute depletion of circulating vasopressin with subsequent hypersensitivity to its effects [37,39]. Because of these theoretical and practical benefits, vasopressin has come into widespread use for shock states, including shock in which myocardial injury plays a contributive role [1-3,12-17]. However, the cardiovascular effect of vasopressin on the injured myocardium has yet to be elucidated.

In this study we found that low-dose vasopressin did not significantly alter arterial blood pressure or cardiac ejection fraction in the uninjured state. This experimental observation correlates with the clinical finding that normotensive patients do not exhibit a physiologic response to low-dose vasopressin [37,39]. In contrast, we found AVP significantly decreased LVEF in a model of ischemia reperfusion. Our model used one hour of LAD ischemia and reproducibly depressed day 1 cardiac ejection fraction by approximately 13% in mice treated with saline infusions (control animals). We compared these saline-infused mice with the standard drug used for cardiogenic shock (DOB) and noted a significant increase in cardiac ejection fraction. This served as both a positive control to assure good absorption of the intra-peritoneal medication as well as a standard of care arm with which to compare cardiac function and mortality. Vasopressin, on the other hand, demonstrated a markedly different effect following LAD I/R than in the intact animal. During the infusion the mean cardiac ejection fraction dropped by 10% when compared with saline, and by 24% compared with baseline. This decrease in cardiac contractility appeared to be through a direct cardiac effect as there was no significant change in either preload (LVEDV) or afterload (arterial blood pressure) due to vasopressin.

Vasopressin had no significant effect on cardiac function in intact mice, while following I/R injury vasopressin was cardio-suppressive. We speculated that this may have resulted from alterations in vasopressin receptor expression as a result of I/R. We found that V1R and OTR were expressed in the heart, while V2R was not detectable. V1R expression was not altered as a result of I/R, while OTR expression was reduced by 40% (Figure 4). This stable expression of V1R combined with decreased OTR expression could result in predominant vasoconstriction in the injured heart, potentially worsening cardiac ischemia and resulting in dysfunction.

In addition to a decline in cardiac contractility, vasopressin resulted in a marked increase in early mortality compared with both saline and DOB-treated mice. The moderate reduction in cardiac ejection fraction and non-statistically significant trend towards a 5 mmHg decrease in blood pressure in the vasopressin-infused group essentially excludes cardiogenic shock as a cause of the excess mortality. Further support for this comes from routine monitoring of the post-operative appearance (grooming, temperature, activity level), with all groups appearing healthy with no evidence of general medical deterioration as would be expected with cardiac insufficiency. Vasopressin and its analogue terlipressin have been reported to induce cardiac arrhythmia (bradycardia and *Torsade de Pointes*) not associated with clear evidence of myocardial infarction [40-45]. Given the generally healthy clinical condition of the mice, we speculate that the majority of deaths may have related to sudden cardiac events (arrhythmia). How might this occur? Vasopressin has been found to block K_ATP _channels in the vascular endothelium, where this reverses vasoplegia in the systemic circulation [8], but may contribute to sudden vasospasm in the coronary circulation [46]. K_ATP _channels expressed on cardiomyocytes are thought to decrease membrane excitability when activated through stress and thus may be key mediators of ischemic tolerance [46]. Increased membrane excitability as a result of vasopressin acting to close K_ATP _channels could increase the risk of arrhythmia.

Limitations of this study include a lack of continuous cardiac and hemodynamic monitoring. Transient changes in afterload may have influenced the extent of ischemic cardiac damage but may not have been detected by our single measurement, while a lack of continuous cardiac rhythm monitoring did not allow us to determine whether arrhythmia was in fact the major cause of death. Other limitations were the lack of quantification of ischemic myocardium as a result of the I/R, and that we used whole left ventricular tissue rather than isolated cardiomyocyte digestion, and were thus not able to assess from which cell type the vasopressin receptors were derived. Therefore, the down-regulation of OTR must be regarded as hypothesis generating rather than a proof of mechanism.

In summary, we found that low-dose vasopressin infusion had no significant cardiovascular effect in normal mice. In contrast, following ischemic injury to the myocardium vasopressin exerted a strong negative inotropic effect on the heart, resulting in a significant decline in cardiac ejection fraction as measured by echocardiogram. This decline was not mediated through changes in left ventricular preload or afterload at the time point assayed and the possibility of a direct cardiac effect is raised. We speculate that I/R, by decreasing OTR expression in the heart, may result in vasopressin-inducing vasoconstriction and cardiac dysfunction in the injured heart.

## Conclusions

AVP infusion significantly depressed the myocardial function in I/R injured model and increased the mortality rate in comparison with SL and DOB. The use of vasopressin may be associated with cardiac suppression in non-vasodilatory shock states involving significant cardiac injury.

## Key messages

• Vasopressin infusion decreases cardiac ejection fraction and increases mortality after I/R injury.

• The decrease in cardiac ejection fraction is not caused by an increase in afterload, but rather through a decrease in cardiac contractility.

• Vasopressin should be used with caution in patients who may have a cardiac component contributing to their shock.

## Abbreviations

ANOVA: analysis of variance; AVP: arginine vasopressin; DOB: dobutamine; I/R: ischemia/reperfusion; LAD: left anterior descending coronary artery; LVEF: left ventricular ejection fraction; LVEDV: left ventricular end diastolic volume; OTR: oxytocin receptors; P2R: purinergic receptors; RT-PCR: real-time polymerase chain reaction; SL: normal saline solution; V1R: vasopressin 1 receptor; V2R: vasopressin 2 receptor; V3R: vasopressin 3 receptor.

## Competing interests

The authors declare that they have no competing interests.

## Authors' contributions

TI drafted the manuscript, performed echocardiography and molecular experiments, and assisted with animal experiments. JB designed the experiments, wrote the manuscript and performed echocardiography. YW performed animal experiments. MM performed molecular experiments. KW designed the experiments and wrote the manuscript. All authors read and approved the final manuscript.
